# State of the art treatment with Impella® in cardiac surgery in Austria

**DOI:** 10.1007/s00508-024-02408-3

**Published:** 2024-09-09

**Authors:** Dominik Wiedemann, Julia Dumfarth, Andreas F. Zierer, Daniel Zimpfer

**Affiliations:** 1 Division of Cardiac Surgery University Hospital St. Pölten, Karl Landsteiner University of Health Sciences, St. Pölten and Krems, Austria; 2 Department of Cardiac Surgery, Medical University of Innsbruck, Innsbruck, Austria; 3 Department of Cardio-Thoracic and Vascular Surgery, Johannes Kepler University Hospital Linz, Linz, Austria; 4 Department of Cardiac Surgery, Medical University of Vienna, Vienna, Austria

**Keywords:** Impella, Mechanical circulatory support, Heart failure, Medium-term cardiac support, Heart recovery

## Abstract

Since 2022, the mechanical left ventricular support system Impella 5.5® has been used in Austria for patients with cardiogenic shock, advanced heart failure, post-cardiotomy and low output syndrome. The surgical insertion of the Impella 5.5 via the subclavian artery or alternatively via the ascending aorta has become an established procedure for medium-term treatment in patients with cardiogenic shock and bridging scenarios, such as bridge to recovery, bridge to left ventricular assist device (LVAD), bridge to decision, and bridge to heart transplant (HTx) in Austria. All Impella left ventricular heart pumps share the common feature of unloading the left ventricle, with the Impella 5.5 achieving a full cardiac output of 5.5 l/min. The stable positioning via transaxillary or transaortic insertion enables rapid extubation and mobilization of patients in the intensive care unit (ICU), leading to a significantly shorter ICU stay. The combined support of Impella 5.5 with venoarterial extracorporeal membrane oxygenation (VA-ECMO) has also proven effective in certain scenarios. Several nonrandomized studies demonstrated the effectiveness and safety of the Impella 5.5 in practice, which have been included in multiple international guidelines. The advantages of the Impella 5.5 in practice include the easy handling with high positional stability, and low complications rates. This article describes the significance of surgical Impella treatment in Austria from the perspective of Austrian clinical experts.

## Introduction

**Fig. 1:**
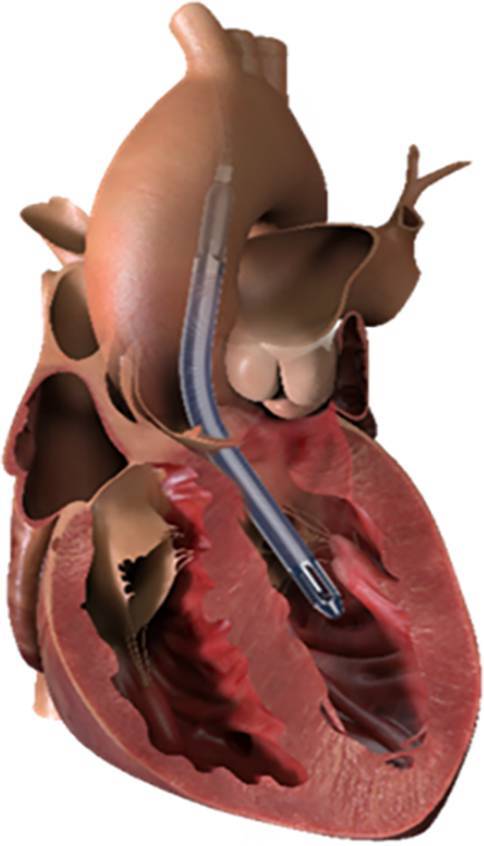
The Impella 5.5. The Impella 5.5 achieves a CO of up to 5.5 l/min and unloads the left ventricle. Figure source: https://www.abiomed.de/ueber-uns/presse-und-news/media-kit

Temporary mechanical circulatory support (tMCS) systems can restore adequate cardiac output (CO) and thus organ perfusion [1–3]. The currently used tMCS systems in Austria include venoarterial extracorporeal membrane oxygenation (VA-ECMO), intra-aortic balloon pump (IABP), and the Impella heart pump (Impella CP®, Aachen, Germany and Impella 5.5, Abiomed, Aachen, Germany) [1]. The Impella is a microaxial blood pump that is either inserted percutaneously via the femoral artery (Impella CP) or inserted surgically via the subclavian artery or ascending aorta (Impella 5.5) and positioned in the left ventricle [4]. The Impella draws blood through an inlet cage and expels it through an outlet area directly into the ascending aorta in a physiological antegrade flow direction, to relieve the left ventricle (Fig. 1; [5]). Unlike VA-ECMO, the Impella reduces left ventricular filling pressures, mechanical cardiac work, and myocardial oxygen consumption. In clinical practice, the Impella 5.5 achieves a CO of up to 5.5 l/min, which corresponds to full CO for most patients. Currently, the Impella 5.5 is approved for a duration of up to 29 days in the European Union [5]. Real-world experience, however, has shown that longer durations (up to over 100 days) are possible [6]. In addition to tMCS systems, permanent invasive MCS systems such as the LVAD are used.

The main indications for the Impella 5.5 include cardiogenic shock, post-cardiotomy cardiogenic shock (PCCS), low cardiac output syndrome (LCOS), and acute decompensated heart failure as well as left ventricular unloading during VA-ECMO (ECMella) (Table 1; [5]). The decision to use the heart pump is based on the expected duration and flow rate of the necessary cardiac support. The Impella 5.5 can serve as medium-term support for the evaluation and preparation of further medical measures (bridge to decision), such as subsequent implantation of a permanent heart support system (bridge to LVAD), a heart transplant (bridge to HTx), or ideally achieving complete native heart recovery (bridge to recovery). This medium-term support provides valuable time for the heart to recover and for the treatment team to develop an optimal patient-specific treatment strategy. Moreover, Impella insertion does not require intrathoracic surgery, which would be a significantly more invasive approach with potential new myocardial damage.

**Table 1:** Common indications for using Impella 5.5 in Austria

Indications for Impella 5.5
*Cardiogenic shock* SCAI Stages C to E Primary therapy or as escalation from another short-term tMCS (IABP, Impella CP or VA-ECMO)
*Post-cardiotomy cardiogenic shock*
*Low cardiac output syndrome*
*Acute decompensated heart failure*
*Advanced heart failure* Bridge to LVAD Bridge to HTx
*Left ventricular Unloading during VA-ECMO (ECMella)*

*tMCS* temporary mechanical circulatory support, *IABP* intra-aortic balloon pump, *VA-ECMO* venoarterial extracorporeal membrane oxygenation, *LVAD* left ventricular assisted device, *HTx* heart transplant

**Fig. 2:**
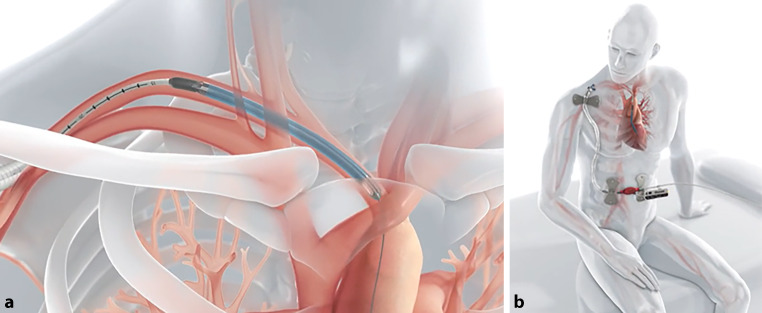
The Impella 5.5 insertion. **a** The Impella 5.5 is inserted by a minimally invasive surgical technique via the subclavian artery. **b** It provides medium-term tMCS for rapid patient mobilization. Figure source: https://www.abiomed.de/ueber-uns/presse-und-news/media-kit

The Impella CP is primarily used percutaneously for rapid stabilization of the patient, with specific support for cardiogenic shock. Escalation to the Impella 5.5 should occur in our experience within a maximum of 48 h to achieve an adequate flow rate, minimize the risk of complications, and enable patient mobilization (Fig. 2; [4]); however, under certain circumstances insertion of an Impella 5.5 as a first-line treatment is also possible. In our practice it has been shown that significantly longer support duration times, beyond the approved duration of 29 days [5], are possible and safe. As such, this would extend the time window for a decision on the necessary treatment for the patient, such as permanent support with an LVAD or HTx.

## Overview of recently published literature

A recent systematic review and meta-analysis focused on adverse events and survival in patients supported by Impella 5.0® (Abiomed, Aachen, Germany) or 5.5 between September 2019 and March 2023 [7]. Of the studies selected for qualitative review, the patient population was heterogeneous with the majority of patients with cardiogenic shock, followed by acute myocardial infarction with cardiogenic shock (AMICS) and PCCS. The pooled survival to hospital discharge was 68% (95% confidence interval, CI 58–78%) and 30-day survival was 65% (95% CI 56–74%). Interestingly, among patients with Impella 5.5, survival to discharge was 78% (95% CI 72–82%) [7]. Overall, these results are consistent with data from an analysis of the National Inpatient Sample Database, which is a representative database of the US population. In this study, the in-hospital mortality rate was approximately 31% in patients who received Impella CP, 2.5® (Abiomed, Aachen, Germany), 5.0 and/or 5.5 [8]. Similarly, an analysis of the quality assurance database in the USA showed a survival rate of 80.5% in patients who were treated with Impella 5.5 [4]. Based on these data and our experience, we speculate that the survival rate would be significantly higher if these critically ill patients were supported with Impella 5.5 rather than the now discontinued Impella 5.0. This should be a focus of future studies.

The Danish German Cardiogenic Shock (DanGer Shock) Trial (NCT 01633502) was the first randomized controlled trial to show a survival benefit for tMCS with short-term support in patients with cardiogenic shock after ST elevation myocardial infarction (STEMI) [9]. Briefly, this study examined the use of Impella CP after STEMI-induced cardiogenic shock versus standard of care treatment without tMCS on top of medical treatment in both arms. While 15.6% of patients in the Impella CP arm required an additional device for mechanical circulatory support (predominantly VA-ECMO) as escalation, 21.0% in the standard care group received a tMCS during the course of treatment. The primary endpoint, death from any cause after 180 days, was significantly reduced in the Impella CP arm compared to standard care (45.8% vs. 58.5%, hazard ratio, HR 0.74: 95% CI 0.55–0.99; *p* = 0.04). Furthermore, the absolute reduction in the risk of death for the Impella CP group of 12.7% results in a number needed to treat of 8. For the combined safety endpoint, significantly more complications occurred in the Impella CP group than in the standard care group (24.0% vs. 6.2%, relative risk, RR 4.74; 95% Confidence intervall, CI 2.36–9.55); however, these higher rates of severe complications, particularly moderate/severe bleeding (21.8% vs. 11.9%, RR 2.06; 95% CI 1.15–3.66), leg ischemia (5.6% vs. 1.1%, RR 5.15; 95% CI 1.11–23.84) and renal replacement therapy (41.9% vs. 26.7%, RR 1.98; 95% CI 1.27–3.09) did not overshadow the benefit of tMCS treatment [9].

## Guidelines and recommendations

Despite the lack of randomized trials, international scientific societies universally recommend the use of the Impella 5.0/5.5 under certain conditions for selected patients in their guidelines and various consensus statements. The Society of Cardiovascular Angiography and Interventions (SCAI) has classified five shock stages in a 2019 consensus statement. According to this, tMCS should be used in shock stages C (classic), D (deteriorating) and E (extreme) within 6 hours [10]. In the 2021 guidelines by the European Society of Cardiology (ESC) on acute and chronic heart failure, the tMCS treatment was raised from a class IIb recommendation/level of evidence C to a class IIa/C without new trial evidence [11]. This is in contrast with IABP, which is no longer recommended for routine use (class III/B) due to the negative IABP-SHOCK II study [12]. In the joint consensus statement of the European Association for Cardio-Thoracic Surgery (EACTS), the Extracorporeal Life Support Organization (ELSO), the Society of Thoracic Surgeons (STS) and the American Association for Thoracic Surgery (AATS) [13] there is a IIb/C recommendation for Impella 5.0 for patients as initial treatment or as joint treatment with VA-ECMO in the presence of severe isolated left ventricular dysfunction. The guidelines of the International Society for Heart and Lung Transplantation (ISHLT) and the Heart Failure Society of America on acute mechanical circulatory support recommend the acute use of MCS devices in the case of SCAI stages C and D of cardiogenic shock with a class I/level B [14]. Nonetheless, mortality remains high in this patient population suggesting refinement and more cohesive guidelines are needed.

With respect to Impella, studies conducted with the Impella 5.0 have been incorporated into guidelines. With the product similarities to Impella 5.0 and further technical enhancements of Impella 5.5, we assume that the same guidance can be applied to use Impella 5.5, although data are needed to confirm that. Furthermore, while a series of single center and multicenter data and various reviews with Impella 5.5 are available [7], randomized clinical trial data in homogeneous patient populations treated with Impella 5.5 are still lacking.

## Discussion

The Impella 5.5 microaxial flow pump achieves adequate circulatory support for acute and chronic heart failure patients by unloading the left ventricle to the extent of the full cardiac output [4]. By reducing the left ventricular pumping work of the native heart, the chance of recovery is preserved, especially in the acute setting, and enables early assessment of the residual state of myocardial function. Moreover, inserting the Impella 5.5 via the subclavian artery or via the ascending aorta, significantly reduces complications that can occur with other tMCS devices that require placement via a femoral approach (e.g. leg ischemia) [15]. Consequently, this insertion technique even enables extubation and mobilization of the patient. In addition, the design of the Impella 5.5 reduces the risk of hemolysis and ensures positional stability [4].

Although initial data from the DanGer Shock Trial [9] are promising, we hypothesize that the immediate approach of an Impella 5.5 treatment or faster escalation from Impella CP to Impella 5.5 may potentially lead to improved outcomes. This speculation is largely derived from the fact that patients in severe cardiogenic shock typically need more potent support, which can be achieved with peak flow of up to 5.5 l/min by the Impella 5.5.

In Austria the ECMella combination treatment is utilized in two ways. First, Impella 5.5 can be employed as an unloading strategy either before or after VA-ECMO implantation to reduce afterload [16]. Second, VA-ECMO can serve as an escalation strategy in cases where the Impella 5.5 was previously inserted, particularly when the patient’s condition necessitates high flow rates and/or oxygenation and/or right heart support [17].

We believe that medium-term support with Impella 5.5 closes a therapeutic gap by enabling the optimization of heart function while waiting for HTx without increasing the risk of complications. As such, the time saved by the medium-term use of an Impella 5.5 enables further treatment strategies to be contemplated (bridge to decision, bridge to LVAD, bridge to HTx), while keeping the option for a complete recovery of the native heart (bridge to recovery).

The use of the Impella 5.5 as a protective post-cardiotomy tMCS (protected cardiac surgery) is an emerging treatment concept across Europe. In cases of mitral valve repair for example, the tMCS device is inserted directly in the operating room. In instances such as these, the patient could be weaned from the heart-lung machine and supported with the Impella 5.5 in the intensive care unit until extubation and beyond, creating new lifesaving opportunities in Austria. Data from the IMpella-Protected cArdiaC surgery Trial in Europe [18] (IMPACT EU, NCT05756751) and Impella in Cardiac Surgery [19] (ImCarS, DRKS00024560) registry are highly anticipated to address the identified evidence gap. More specifically, the IMPACT EU trial is investigating the efficacy and safety of perioperative Impella 5.5 use in high-risk patients with severe left ventricular dysfunction who require cardiac surgery with cardiopulmonary bypass. In this prospective non-randomized study 123 patients are expected to be enrolled. The primary endpoint analyzes the rate of post-cardiotomy cardiac failure at hospital discharge, and secondary, all-cause mortality and stroke [18]. In the case of the ImCarS registry, the surgical performance quality and clinical management of patients supported with tMCS devices such as the Impella 5.0/5.5 or Impella RP® (Abiomed, Aachen, Germany; for right heart support) with or without VA-ECMO will be assessed. The outcome measures of interest are survival to discharge or next treatment and all-cause mortality at 1 month, interim (between 3 and 9 months) and 1 year post-procedure [19].

## Conclusion

In practice, Impella 5.5 closes the gap in care between short-term tMCS treatment (e.g. Impella CP) and long-term treatment with a permanently implanted LVAD or a donor heart. With an approved duration of ≤ 29 days, which is exceeded internationally and in Austrian centers due to the low complication rate, the Impella 5.5 represents a medium-term treatment option. In combination treatment, such as ECMella, Impella 5.5 facilitates weaning from VA-ECMO, extubation, and earlier mobilization of patients, which may potentially save ICU capacity. Consequently, the surgically inserted Impella 5.5 is the standard of care for medium-term treatment in Austria for patients with cardiogenic shock and for bridging scenarios, such as bridge to recovery, bridge to LVAD, bridge to decision, and bridge to HTx.
